# Cutaneous *Legionella longbeachae* Infection in Immunosuppressed Woman, United Kingdom

**DOI:** 10.3201/eid2108.140828

**Published:** 2015-08

**Authors:** Daniel Grimstead, David Tucker, Kathryn Harris, Deborah Turner

**Affiliations:** South Devon Healthcare Foundation Trust, Torquay, UK (D. Grimstead, D. Tucker, D. Turner);; Great Ormond Street Hospital, NHS Foundation Trust, London, UK (K. Harris)

**Keywords:** legionellosis, Legionella, chronic lymphocytic leukemia, immunosuppression, bacteria, United Kingdom, Legionella longbeachae, cutaneous

## Abstract

We report a rare case of cutaneous *Legionella longbeachae* infection in a patient receiving long-term corticosteroids for immune thrombocytopenia. Such infections cannot be identified by using *Legionella* urinary antigen testing but are commonly seen after exposure to commercial potting compost, particularly in immunocompromised patients.

A 70-year-old woman in whom stage A0 chronic lymphocytic leukemia had been diagnosed in 2004 by immunophenotyping was monitored with a watch-and-wait strategy for 8 years until she started treatment for anemia and splenomegaly. She was given 4 cycles of fludarabine, cyclophosphamide, and rituximab until her illness went into clinical remission.

Twelve months later, the woman sought treatment for extensive purpura. She had a platelet count of 1 × 10^9^/mL (reference range 150–400 × 10^9^/mL) but an otherwise unremarkable complete blood count, peripheral blood smear, and bone marrow aspirate and trephine biopsy result. Immune thrombocytopenia was diagnosed, and 1 mg/kg of oral prednisolone daily was initiated, to which the illness responded well. Over 8 weeks, steroids were weaned to 20 mg once daily. During this time, the woman had a colonoscopy to investigate 6 weeks of persistent diarrhea. Cultures of fecal samples and multiple colonic biopsies were unremarkable.

During a routine outpatient review in August 2013, three discrete erythematous nodules were found on the ventral surface of her right forearm. The lesions were tender and nonpurulent, contained no punctum, and were distinct and separate from the site where an intravenous cannula had been placed for her colonoscopy. She had no history of tick or animal bite, recent foreign travel, or trauma. Her diarrheal illness had resolved before this review. Cellulitis was presumptively diagnosed, and the woman was started on a course of oral flucloxacillin.

Two weeks later, she was admitted to the hospital with worsening pain and swelling of the right forearm. She remained systemically well. Laboratory testing indicated a leukocyte count of 9.6 × 10^9^ cells/L (reference range 4.0–11.0 × 10^9^ cells/L), with neutrophils 8.7 × 10^9^ cells/L (reference range 1.8–7.5 × 10^9^ cells/L), lymphocytes 0.4 × 10^9^ cells/L (reference range 1.5–4.0 × 10^9^ cells/L), monocytes 0.4 × 10^9^ cells/L (reference range 0.2–1.0 × 10^9^ cells/L), eosinophils 0.1 × 10^9^ cells/L (reference range 0–0.4 × 10^9^ cells/L), and basophils 0 × 10^9^ cells/L (reference range 0–0.4 × 10^9^ cells/L). C-reactive protein level was 17 mg/L. She was treated empirically with intravenous flucloxacillin.

Initial blood culture results were negative. Cultures of wound swab samples were negative for methicillin-resistant *Staphylococcus aureus* but showed evidence of gram-negative bacilli. Results from urinary *Legionella* and pneumococcal antigen tests were negative. Surgical review prompted incision and drainage of the lesions, the largest of which was 4 cm × 5 cm (Figure). Histopathologic examination of the lesions showed only granulation tissues; no malignant cells were seen.

**Figure:**
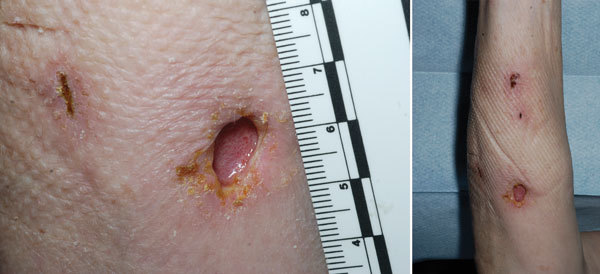
Forearm lesion after incision and drainage in immunosuppressed woman with cutaneous *Legionella longbeachae* infection, United Kingdom.

Lack of clinical improvement prompted a change in antimicrobial drug therapy to intravenous tazocin and clindamycin. Results from serial bacterial cultures of blood, feces, and tissue failed to yield further positive results. Results from PCR for herpes simplex virus types 1 and 2 and varicella zoster virus were negative. Serum β-D-glucan was elevated at 262 pg/mL (reference range <80 pg/mL); however, the patient did not receive antifungal treatment because of the lack of clinical suspicion of fungal infection and the test’s high false-positive rate.

A dermatology review suggested that the lesions represented a sporotrichoid lymphocutaneous infection, possibly caused by an atypical mycobacterium. Results from an extended culture for mycobacterium, auramine stains for acid-fast bacilli, and further histopathologic staining for fungal hyphae were negative. Despite antimicrobial drug therapy, the lesions persisted for 2 weeks; repeat incision and drainage was performed.

A pus sample was sent to Great Ormond Street Hospital (London, UK) for PCR. Species-specific real-time PCR for *S. aureus* and *S. pyogenes* were negative. Subsequent analysis by broad-range 16S rDNA PCR (*1*) gave a strongly positive result, and the amplicon sequence exactly matched 16S rDNA sequences from 2 *L. longbeachae*–type strains, leading to a diagnosis of cutaneous infection with *L. longbeachae.*

The woman was started on a 6-week course of triple antimicrobial drug therapy with ciprofloxacin, azithromycin, and rifampin. By her 3-month follow-up, her lesions had resolved completely. Although she was not a keen gardener, she reported that, a week before her lesions appeared, she had handled a leaking potted plant without thoroughly washing her hands afterward.

*Legionella longbeachae* was first described in Long Beach, California, USA, in 1981; it was isolated from respiratory tract specimens from 4 patients with pneumonia (*2*). A second serogroup was identified later that year (*3*). Reported cases of *L. longbeachae* infection are rare in Europe. However, in New Zealand, Australia, and Japan, they are as common as infections with *L. pneumophila* and often cause legionellosis and Pontiac fever, a nonpneumonic, self-limiting illness characterized by influenza-like symptoms (*4*,*5*). Only testing of urinary antigens can identify *L. pneumophila* serogroup 1, whereas *L. longbeachae* can be detected by serologic testing, culture, or PCR (*6*).

Unlike infections with *L. pneumophila*, which have been linked to water systems in the built (i.e., human-created) environment, infections with *L. longbeachae* are most commonly associated with the use of commercial potting compost (*4*,*7*,*8*). *L. longbeachae* was first isolated from potting mix after an outbreak of infections in South Australia in 1989 (*9*). Since then, outbreaks have been linked to the use of potting compost in Australia, New Zealand, Japan, the Netherlands, and most recently Scotland, where 4 cases of *L. longbeachae* infection were confirmed during 2008–2009 (*4*,*5*,*7*,*10*). A 2013 study of compost in the United Kingdom found that 15 of the 24 samples tested contained *Legionella* species, of which 4 were identified as *L. longbeachae* serogroup 1 (*11*).

A 2006 case–control study (*7*) showed that risk factors that predicted *L. longbeachae* infection included poor hand hygiene after gardening and proximity to dripping hanging flower pots, the latter indicating that ingestion might be an alternate possible route of transmission to aerosolization (*4*,*7*). These risk factors suggest a possible route of infection for the patient reported here; she reported handling a large, leaking potted plant and admitted to poor hand hygiene before meals. However, *L. longbeachae* cannot be confirmed as the source of her infection because her potting soil was not tested. An alternative route of infection would be through the site of the cannula that was inserted for her colonoscopy. Other reported risk factors include smoking, preexisting respiratory disease, and immunosuppression (*4*,*6*,*7*,*12*). The primary host defense mechanism in *L. longbeachae* infection is cell-mediated immunity, depression of which through the use of corticosteroids or immunosuppressive drugs may predispose patients to legionellosis. A 2007 report describes a case of *L. longbeachae* pneumonia after corticosteroid therapy for chronic immune thrombocytopenia (*6*).

The clinical picture in *L. longbeachae* infection is typically similar to that of infection with *L. pneumophila*; however, as in this case, variations have been reported. A 2012 case report described a patient in whom *L. longbeachae* endocarditis developed 6 months after a bioprosthetic aortic valve replacement (*13*). Several cases of cutaneous infection secondary to *Legionella* species have been reported (*14*); 7 of the 13 confirmed cases occurred in immunocompromised patients. Because so many cases occurred in immunocompromised patients, we recommend use of broad-range 16S rDNA PCR to detect *L. longbeachae* in immunosuppressed patients with respiratory or influenza-like symptoms who report a history of exposure to commercial potting compost.
